# Comparison of epicardial adipose tissue volume quantification between ECG-gated cardiac and non-ECG-gated chest computed tomography scans

**DOI:** 10.1186/s12872-022-02958-2

**Published:** 2022-12-13

**Authors:** Yuancheng Xu, Stanislau Hrybouski, D. Ian Paterson, Zhiyang Li, Yulong Lan, Lin Luo, Xinping Shen, Lingyu Xu

**Affiliations:** 1 Department of Urology, The University of Hong Kong-Shenzhen Hospital, Shenzhen, Guangdong China; 2 Neuroscience and Mental Health Institute, University of Alberta, Edmonton, Alberta Canada; 3 Department of Cardiology, Mackenzie Health Science Centre, Faculty of Medicine and Dentistry, University of Alberta, Edmonton, Alberta Canada; 4 Department of General Surgery, the Second Affiliated Hospital of Shantou University Medical College, Shantou, Guangdong China; 5 Department of Cardiology, the Second Affiliated Hospital of Shantou University Medical College, Shantou, Guangdong China; 6 Department of Radiology, The University of Hong Kong-Shenzhen Hospital, Shenzhen, Guangdong China; 7 University of Alberta, 2C2, Mackenzie Health Science Centre, 8440 - 112 St, Edmonton, Alberta T6G 2B7 Canada

**Keywords:** Epicardial adipose tissue, Chest Computed tomography, Tube voltage, Contrast enhancement, Slice thickness

## Abstract

**Background:**

This study investigated accuracy and consistency of epicardial adipose tissue (EAT) quantification in non-ECG-gated chest computed tomography (CT) scans.

**Methods:**

EAT volume was semi-automatically quantified using a standard Hounsfield unit threshold (− 190, − 30) in three independent cohorts: (1) Cohort 1 (*N* = 49): paired 120 kVp ECG-gated cardiac non-contrast CT (NCCT) and 120 kVp non-ECG-gated chest NCCT; (2) Cohort 2 (*N* = 34): paired 120 kVp cardiac NCCT and 100 kVp non-ECG-gated chest NCCT; (3) Cohort 3 (*N* = 32): paired non-ECG-gated chest NCCT and chest contrast-enhanced CT (CECT) datasets (including arterial phase and venous phase). Images were reconstructed with the slice thicknesses of 1.25 mm and 5 mm in the chest CT datasets, and 3 mm in the cardiac NCCT datasets.

**Results:**

In Cohort 1, the chest NCCT-1.25 mm EAT volume was similar to the cardiac NCCT EAT volume, while chest NCCT-5 mm underestimated the EAT volume by 7.5%. In Cohort 2, 100 kVp chest NCCT-1.25 mm were 13.2% larger than 120 kVp cardiac NCCT EAT volumes. In Cohort 3, the chest arterial CECT and venous CECT dataset underestimated EAT volumes by ~ 28% and ~ 18%, relative to chest NCCT datasets. All chest CT-derived EAT volumes were similarly associated with significant coronary atherosclerosis with cardiac CT counterparts.

**Conclusion:**

The 120 kVp non-ECG-gated chest NCCT-1.25 mm images produced EAT volumes comparable to cardiac NCCT. Chest CT EAT volumes derived from consistent imaging settings are excellent alternatives to the cardiac NCCT to investigate their association with coronary artery disease.

**Supplementary Information:**

The online version contains supplementary material available at 10.1186/s12872-022-02958-2.

## Introduction

The epicardial adipose tissue (EAT)—located between the outer wall of the myocardium and the visceral pericardium—has proatherogenic effect on the coronary arteries via secretion of pro-inflammatory cytokines [1, 2]. Traditionally, EAT volumes have been measured using electrogram-gated (ECG-gated) cardiovascular non-contrast computed tomography (NCCT) images [1, 3, 4]. More recently, non-ECG-gated chest CT images have been incrementally used to quantify EAT volume [5–8], which provide an appealing alternative to ECG-gated cardiac NCCT because of their widespread use in clinical practice [9]. Examining EAT volumes using chest CT may enable early stratification of cardiovascular risk for patients undergoing chest CT scans [8, 10]. Currently, cardiac NCCT acquisitions with ECG gating, tube voltage of 120 kVp, and a slice thickness of 3 mm are the standard for quantifying EAT volume [3, 11, 12]. The effects of chest CT acquisition and reconstruction parameters—i.e., presence vs. absence of ECG gating or contrast agents, differences in tube voltage and slice thickness, relative to cardiac CT scans—on calculated EAT volumes are still poorly understood.

Chest CT does not use ECG gating, making the heart and EAT pool susceptible to artifacts from cardiac motion. Furthermore, without ECG gating, chest CT, unlike cardiac CT, is unable to acquire images at fixed points of the cardiac cycle [13]. Tube voltage substantially affects tissue attenuation in the CT images [14]. Given that EAT volume quantification is based on a radiodensity range (e.g., − 190 to − 30 HU) [3, 4, 7, 15–17], understanding impacts of tube voltage on EAT volumes derived from chest CT scans is vital for measurement consistency with cardiac CT scans. The reconstructed slice thickness is commonly 3 mm in the cardiac NCCT image, but chest CT images are most commonly reconstructed with 1 mm, 1.25 mm, 2.5 mm and 5 mm [5, 8, 18, 19]. Thus, the effects of chest CT slice thickness on EAT volumes need to be investigated further. Finally, contrast enhancement in the arterial phase has been shown to underestimate EAT volume derived from cardiac contrast-enhanced CT images (CECT) compared to those derived from cardiac NCCT images using the same radiodensity thresholds [11, 17]. To date, no study has investigated the effect of contrast agents on non-ECG-gated chest CT-derived EAT and the difference of contrast enhancement between arterial phase and venous phase on EAT volume quantification.

This study aims to investigate the consistency and accuracy of studying EAT volumes using a number of commonly acquired chest CT datasets. Non-ECG-gated chest CT EAT volumes were then compared to those that were obtained using the standard setup for EAT quantification—120 kVp ECG-gated cardiac NCCT. Additionally, we evaluated the effect of tube voltage, slice thickness and contrast enhancement in arterial phase and venous phase on EAT volume measurement in chest CT.

## Materials and methods

### Patient recruitment

We retrospectively reviewed chest CT dataset and cardiac CT dataset between 2012 and 2019 from the University of Hong Kong-Shenzhen Hospital clinical database. The inclusion criterions of Cohort 1 and Cohort 2 included: (1) The subject underwent both cardiac CT and chest CT; (2) The cardiac CT included at least cardiac NCCT and the chest CT at least included chest NCCT; (3) To minimize the effect of time on EAT volume, time interval between paired CT scans was restricted such that the two scans were acquired no more than two weeks apart. Finally, we were able to identify 83 patients who underwent paired non-ECG-gated chest NCCT and ECG-gated cardiac NCCT scans. For non-ECG-gated chest NCCT vs. chest CECT in the arterial phase and venous phase comparisons in Cohort 3, all datasets were acquired in the same session with the other imaging parameters fixed, except the use of contrast agent, in January 2019. This study was approved by the Medical Research Ethics committee of the University of Hong Kong-Shenzhen Hospital.

The imaging protocol is summarized in Table 1. Two slice thicknesses of 1.25 mm and 5.0 mm were used separately for chest CT image reconstruction in all patients and used for comparisons in all cohorts. Cohort 1 consisted of 49 patients who underwent paired ECG-gated cardiac NCCT and non-ECG-gated chest NCCT scans using the same tube voltage 120 kV. Cohort 2 consisted of 34 patients who underwent paired cardiac NCCT (120 kVp) and non-ECG-gated chest NCCT (100 kVp). Lastly, the effects of contrast enhancement on EAT volumes were quantified using paired non-ECG-gated chest NCCT vs. chest arterial CECT vs. chest venous CECT in Cohort 3 (*N* = 32).

**Table 1:** CT acquisition and reconstruction parameters in three cohorts

Paired CT dataset	Cohort 1 (*N* = 49)		Cohort 2 (*N* = 34)		Cohort 3 (*N* = 32)		
	ECG-gated Cardiac NCCT	Non-ECG-gated Chest NCCT	ECG-gated Cardiac NCCT	Non-ECG-gated Chest NCCT	Non-ECG-gated Chest NCCT	Non-ECG-gated Chest arterial CECT	Non-ECG-gated Chest venous CECT
ECG gate	Yes	No	Yes	No	No	No	No
Field of view	150 ~ 170 × 150 ~ 170 mm	360 × 300 ~ 360 mm	150 ~ 170 × 150 ~ 170 mm	360 × 300 ~ 360 mm	360 × 300 ~ 360 mm	360 × 300 ~ 360 mm	360 × 300 ~ 360 mm
Matrix size	512 × 512	512 × 512	512 × 512	512 × 512	512 × 512	512 × 512	512 × 512
Tube voltage (kVp)	120	120	120	100	120	120	120
Contrast given	No	No	No	No	No	Yes, arterial phase	Yes, venous phase
Collimation (mm)	128 × 0.6	128 × 0.625	128 × 0.6	128 × 0.625	128 × 0.625	128 × 0.625	128 × 0.625
Gantry rotation time (ms)	330	350	330	350	350	350	350
Pitch	0.24	1.2	0.24	1.2	1.2	1.2	1.2
Reconstructed slice thickness (mm)	3	1.25 and 5	3	1.25 and 5	1.25 and 5	1.25 and 5	1.25 and 5

ECG, electrocardiogram, NCCT, non-contrast computed tomography, CECT, contrast-enhanced computed tomography

### Cardiovascular CT protocol and analysis

Our ECG-gated cardiac NCCT images were acquired using a 64 detector-row CT scanner (Somatom Definition AS, Siemens, Germany) with the following parameters (Table 1): retrospective ECG gating, tube voltage = 120 kVp, collimation = 128 × 0.6 mm, gantry rotation time = 330 ms, pitch = 0.24, field of view = 150 ~ 170 mm × 150 ~ 170 mm, matrix size = 512 × 512. Z-axis coverage extended from the pulmonary artery bifurcation to the ventricular apex. Reconstruction was performed at 70% of the RR wave, and images were reconstructed with a 3-mm slice thickness (+ 1.5-mm interslice gap) for cardiac NCCT, and a 0.6-mm slice thickness (+ 0.3-mm interslice gap) for cardiac CECT using soft-tissue convolution kernel (B35f). To maintain heart rate at or below 70 bpm, patients were pre-treated with oral metoprolol, as necessary. After the cardiac NCCT scans, patients were intravenously injected 75 ml Iopamidol 370 (Bracco, Italy) with an injection rate of 4–5 ml/s.

Coronary calcium score (CAC) was quantified in the cardiac NCCT image using by the standard Agatston method [20]. The risk of CAD was graded based on the total CAC, 0 for no evidence of CAD, 1–10 for minimal risk, 11–100 for mild risk, 101–400 for moderate risk, > 400 for severe risk. Then Moderate to severe evidence of CAD was defined by total CAC > 100 [21, 22].

All coronary arteries with a diameter > 1.5 mm were evaluated via the longitudinal, transverse and oblique planes reconstructed by the multiplanar reconstructions, curved reconstructions, and thin-slab maximum intensity techniques. Obstructive coronary artery disease (CAD)stenosis was defined as the presence of ≥ 1 coronary lesion with ≥ 50% stenosis measured as the target luminal diameter divided by the maximal luminal diameter at the proximal end of the target lesion [23].

### Chest CT protocol

Chest CT images were acquired using a 64 detector-row CT scanner (LightSpeed VCT, GE, USA) with following parameters (Table 1): collimation = 128 × 0.625 mm, gantry rotation time = 350 ms, pitch = 1.2, field of view = 360 mm × 300 ~ 360 mm, matrix size = 512 × 512. The tube voltage was 120 kVp in Cohorts 1 and 3, and 100 kVp in Cohort 2. Images were reconstructed as 1.25-mm thick slices (+ 1-mm interslice gap) and 5-mm thick slices (+ 5-mm interslice gap), respectively. A soft-tissue convolution kernel of B31f was used. Participants in Cohort 3 first underwent non-ECG-gated chest NCCT image acquisition and then, to acquire the chest CECT image, they were intravenously administered a bolus of contrast medium (Iopromide 370, Bayer Schering Pharma AG; dosage = 1 ml/kg body weight, rate = 3 ml/s) followed by a flush of normal saline (30 ml). Then the chest CECT image was acquired at the arterial phase and venous phase, respectively. Coverage in all chest CT datasets extended from the thoracic inlet to the upper abdomen, at a minimum.

### Epicardial adipose tissue volume quantification

**Fig. 1:**
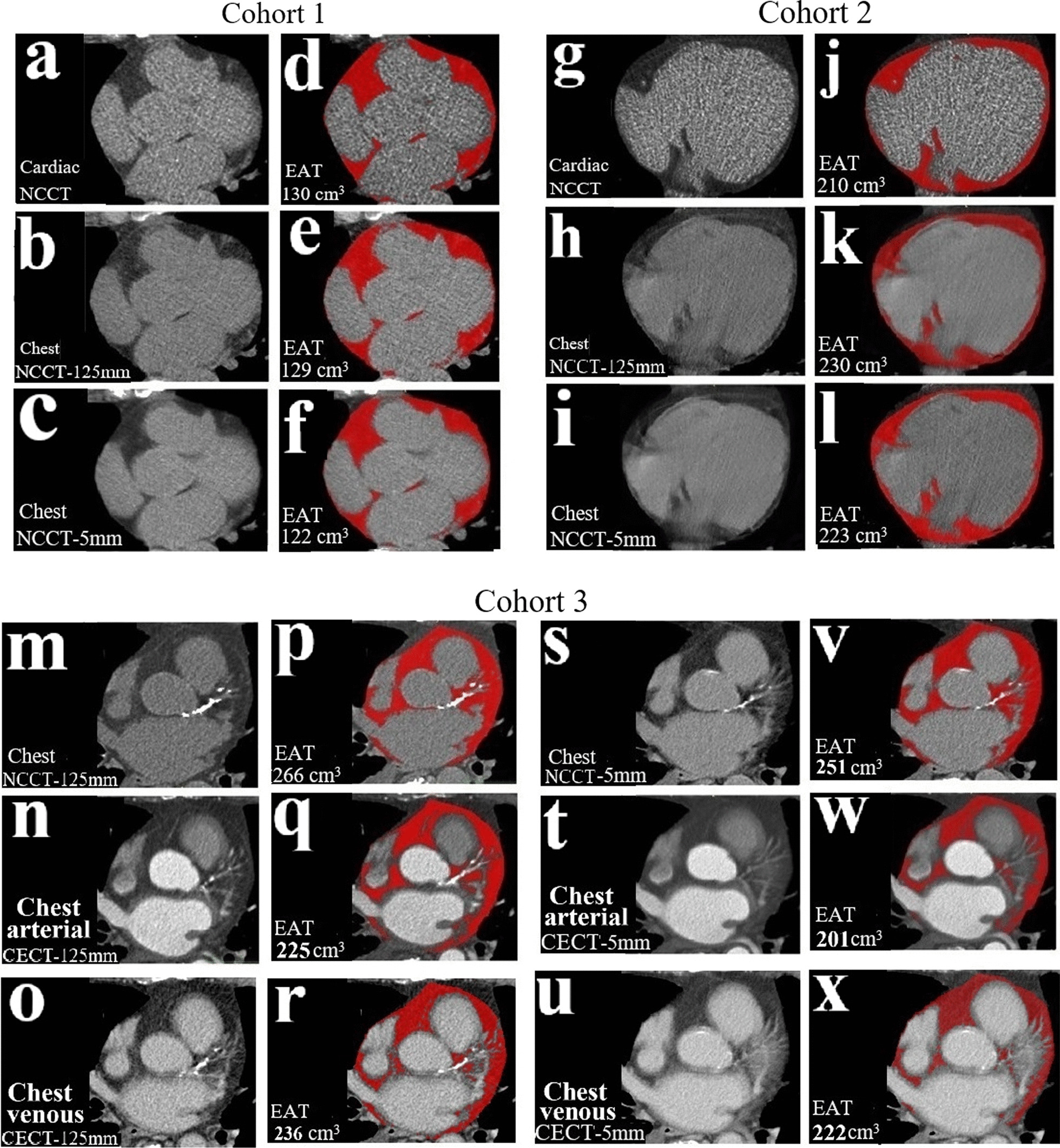
**Examples of EAT volume quantification in three cohorts**–Examples of measuring and comparing EAT volumes at the same slice in paired CT datasets of three cohorts. Each cohort includes CT images in left panel and EAT highlighted in red in the right panel which was semi-automatically quantified using radiodensity threshold of − 190, − 30 HU. Panel (**a**–**f**): Cohort 1, ECG-gated cardiac NCCT (120 kVp) vs. non-ECG-gated chest NCCT-1.25 mm (120 kVp) & chest NCCT-5mm (120 kVp); Panel (**g**–**l**): Cohort 2, ECG-gated cardiac NCCT (120 kVp) vs. non-ECG-gated chest NCCT-1.25 mm (100 kVp) vs chest NCCT-5mm (100 kVp); Panel (**m**–**x**): Cohort 3, non-ECG-gated chest NCCT-1.25 mm (120 kVp) vs. chest CECT-1.25 mm in arterial phase (120 kVp) & chest CECT-1.25 mm in venous phase (120 kVp), and non-ECG-gated chest NCCT-5mm (120 kVp) vs. chest CECT-5mm in arterial phase (120 kVp) & chest CECT-5mm in venous phase (120 kVp). Abbreviation: EAT = epicardial adipose tissue, see others in Table 1

The EAT volume was semi-automatically quantified by a single interpreter (LX) using freely-available a dedicated image analysis software (ITK-SNAP version 3.6.0) [24]. A widely used radiodensity threshold (− 190, − 30) HU [3, 4, 7, 15–17] was set by using in-house MATLAB code and was applied to all cardiac and chest CT images. Longitudinally, EAT quantification began from the bifurcation of the pulmonary artery and ended at the level of the left ventricular apex. The contour of the pericardium was semi-automatically traced on every fifth slice. The segmented pericardial contours were interpolated to the remaining slices and were carefully adjusted if necessary. Finally, the EAT volume and radiodensity were automatically computed. (Fig. 1).

### Statistics

Statistical analyses were performed in STATA (Version 16.0; StataCorp LP, College Station, Texas, USA). Continuous variables were presented as mean ± standard deviation or median (25th, 75th percentile), as appropriate. Categorical variables were expressed as frequencies and percentages. Paired t-tests were used to evaluate the statistical significance of EAT volumes derived from the targeted chest CT imaging data and the reference CT image (i.e., cardiac NCCT in Cohort 1 and 2, chest NCCT in Cohort 3). One-way analysis of variance and Chi-squared tests were used to compare sample characteristics among cohorts. Shapiro–Wilk tests were used to test for normality violations. Bland–Altman analyses were used for assessing the agreement of EAT measurements between different CT scans. We evaluated the value of EAT volumes from different image datasets predicting the presence of obstructive CAD, and moderate to severe evidence of CAD defined by CAC score > 100, respectively, by computing the area under the curve (AUC) of the receiver operating characteristic (ROC) curve through logistic regression, and the equality of AUCs of the same patient cohort was tested. Finally, power analysis, by comparing two correlated means in one sample with α level 0.05 and a two-sided test, was performed based on our result and sample size. *P-*values less than 0.05 were used as a measure of significance in all tests.

## Results

The demographic data and disease history of three cohorts are summarized in Table 2. Mean age for the entire sample was 62 years with 64% males. Twenty (41%) patients in Cohort 1 and 12 (35%) patients in Cohort 2 were diagnosed as coronary artery disease from the cardiac CT images. The time interval between chest CT and cardiac CT scans was 6 ± 4 days. The clinical indications of the CT scans were summarized in Additional file 1: Table S1.

**Table 2:** Basic characteristics for three cohorts

	Overall (*N* = 115)	Cohort 1 (*N* = 49)	Cohort 2 (*N* = 34)	Cohort 3 (*N* = 32)	*p* value
Age (years)	62 ± 11	64 ± 11	57 ± 9	68 ± 9	< 0.001
Male (%)	74 (64%)	30 (61%)	22 (65%)	22 (69%)	0.79
Body mass index (kg/m^2^)	24.7 ± 3.8	24.9 ± 3.6	25.2 ± 4.0	23.7 ± 3.8	0.26
Hypertension	43 (37%)	18 (37%)	11 (34%)	14 (44%)	0.67
Diabetes mellitus	17 (15%)	5 (10%)	10 (29%)	2 (6%)	0.02
Smoking	16 (14%)	8 (16%)	1 (3%)	7 (22%)	0.07
Hyperlipidemia	28 (24%)	11 (22%)	11 (32%)	6 (19%)	0.40
Chronic obstructive pulmonary disease	20 (17%)	9 (18%)	2 (6%)	9 (28%)	0.04
Obstructive coronary artery disease	32 (39%)	20 (41%)	12 (35%)		0.61
Total coronary calcium score	20 (0, 82)	38 (0, 118)	14.1 (0, 46)		0.08

### EAT volume in Cohort 1

**Fig. 2:**
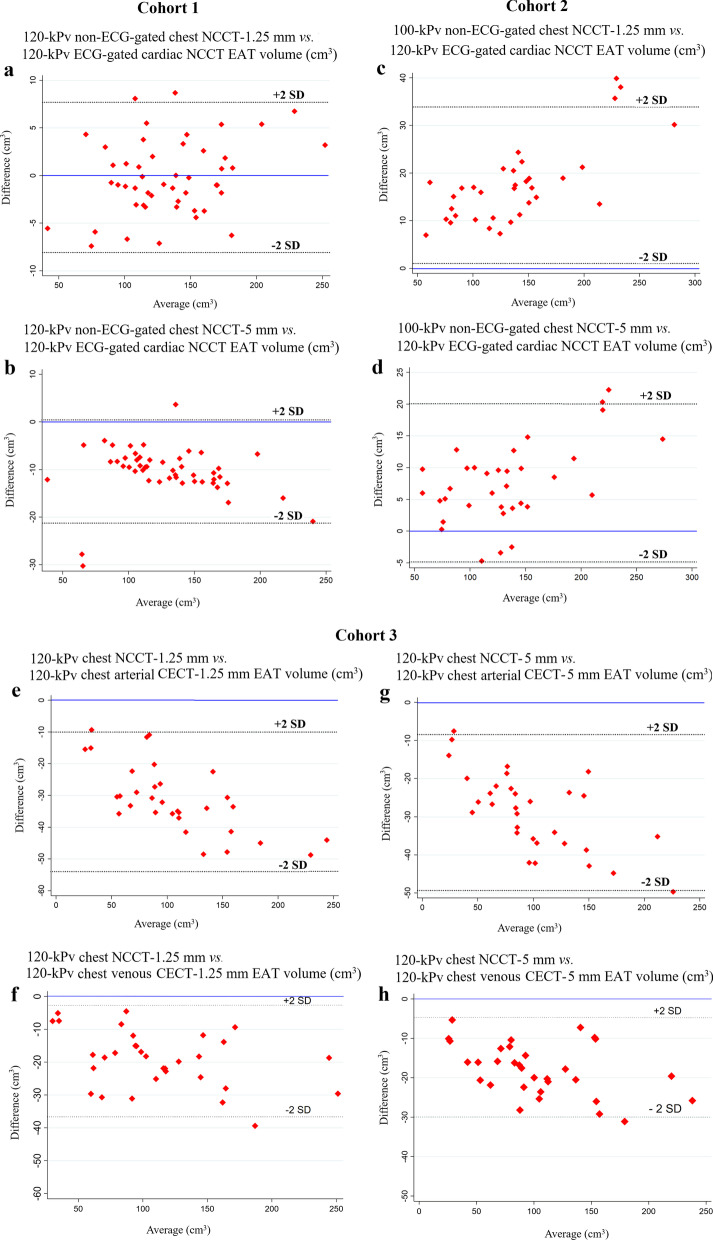
**Bland-Altman plots of assessing the agreement of EAT volume measurements in three cohorts**–Comparison of EAT volumes derived from ECG-gated cardiac NCCT (120 kVp) with non-ECG-gated chest NCCT and effects of contrast agent and slice thicknesses on EAT volume quantification in non-ECG-gated chest CT. In Cohort 1 and Cohort 2, EAT volume derived from cardiac NCCT (referent) vs. non-ECG-gated chest NCCT-1.25 mm (Panel **a** & **c**), and vs. chest NCCT-5 mm (Panel **b** & **d**) images are assessed in Bland-Altman plots for the measurement agreement. In Cohort 3, EAT volume derived from non-ECG-gated chest NCCT-1.25 mm (referent) vs. arterial CECT-1.25 mm (Panel **e**), and vs. venous CECT-1.25 mm (Panel **f**), as well as EAT volume derived from chest NCCT-5 mm (referent) vs. arterial CECT-5 mm (Panel **g**), and vs. venous CECT-5 mm (Panel **h**) are assessed in Bland-Altman plots. Abbreviation: see Table 1

**Fig. 3:**
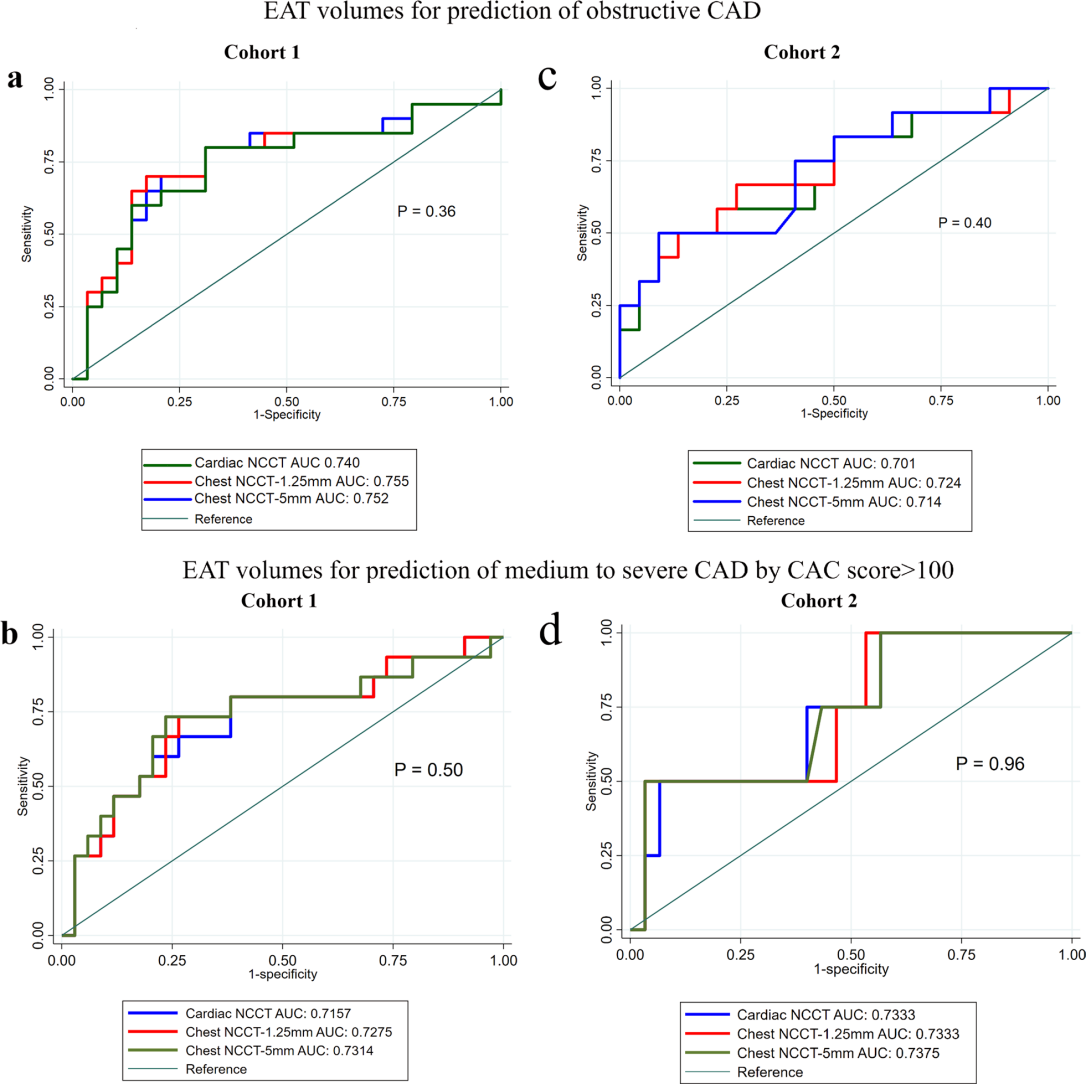
**ROC analysis comparing the value of EAT volumes from different CT images in predicting CAD**–Comparison of the value of cardiac NCCT (referent) vs. non-ECG-gated chest NCCT-1.25 mm vs. chest NCCT-5 mm, with the same tube voltage 120 KV in Cohort 1,in predicting the presence of obstructive CAD (Panel **a**) and severe evidence of CAD by total CAC > 100 (Panel **b**); Comparison of the value of cardiac NCCT (referent) vs. non-ECG-gated chest NCCT-1.25 mm-100 KV vs. chest NCCT-5 mm-100 KV of Cohort 2 in predicting the presence of obstructive CAD (Panel **c**) and severe evidence of CAD by total CAC > 100 (Panel **d**). Abbreviation: AUC = Area under the curve, ROC = receiver operating characteristic, CAD = coronary artery disease, CAC = coronary calcium score, EAT = epicardial adipose tissue. others see Table 1

Compared to EAT volume derived from the reference image (ECG-gated cardiac NCCT image, 120 kV, 3-mm slice thickness), the non-ECG-gated chest NCCT-1.25 mm EAT volumes were similar (133.5 ± 41.3 vs. 133.7 ± 40.2 cm^3^, *p* = 0.74, see Table 3 and Fig. 2), while the nongated chest NCCT-5 mm images produced EAT volume that were consistently lower (123.2 ± 39.6 vs. 133.7 ± 40.2 cm^3^, *p* < 0.001). The EAT radiodensity for the chest NCCT-1.25 mm scans (− 79.4 ± 7.6 HU) was similar to that of the cardiac NCCT (− 78.0 ± 6.3 HU), but lower (i.e., more negative) than that of the chest NCCT-5 mm scans (− 74.4 ± 6.7 HU). Both nongated chest NCCT EAT volumes exhibit similar predicting value of obstructive coronary stenosis (AUC of ROC: 0.755 vs. 0.751 vs. 0.740, *p* = 0.36, Fig. 3a) and moderate to severe CAD defined by CAC score > 100 (AUC of ROC: 0.7275 vs. 0.7314 vs. 0.7157, *p* = 0.50, Fig. 3b) with cardiac NCCT EAT volumes. The nongated chest NCCT-5 mm EAT volumes were lower (Δ = − 9.4 (− 11.8, − 7.3) cm^3^, Δ% = − 7.3 (− 8.6, − 5.7) %, *p* < 0.001) than the chest CT-1.25 mm EAT volumes.

**Table 3:** Comparison of EAT volumes in three cohorts

Cohort 1, *N* = 49 (Time interval of 2 CT scans: < 2 weeks)				Cohort 2, *N* = 34 (Time interval of 2 CT scans: < 2 weeks)		
	Tube voltage: 120 KPV	Tube voltage: 120 KPV		Tube voltage: 120 KPV	Tube voltage: 100 KPV	
	Reference: Cardiac NCCT	Chest NCCT-1.25 mm	Chest NCCT-5 mm	Cardiac NCCT	Chest NCCT-1.25 mm	Chest NCCT-5 mm
EAT volume (cm^3^)	133.7 ± 40.2	133.5 ± 41.3	123.2 ± 39.6	129.6 ± 50.6	147.0 ± 56.6	137.2 ± 53.9
∆Volume, cm^3^ (%) (vs. Reference)	N/A	− 0.9 (− 3.1, 2.6); − 0.6 (− 2.3, 2.6)%	− 9.6 (− 12.5, − 7.4); − 7.5 (− 9.0, − 6.2)%	N/A	16.9 (11.1, 20.5); 13.2 (10.5, 17.0)%	6.9 (3.8, 10.0); 5.8 (2.9, 8.8)%
*p*-value 1 (vs. Reference)	N/A	0.74	< 0.001	N/A	< 0.001	< 0.001
EAT radiodensity (HU)	− 78.1 ± 6.3	− 79.4 ± 7.6	− 74.5 ± 6.7	− 78.0 ± 4.7	− 83.9 ± 6.1	− 79.5 ± 5.5
*p* -value 2 (vs. Reference)	N/A	0.10	< 0.001	N/A	< 0.001	0.021

Cohort 3, *N* = 32						
	Reference: Chest NCCT-1.25 mm	Chest CECT- 1.25 mm-arterial	Chest CECT-1.25 mm-venous	Reference: Chest NCCT-5 mm-	Chest CECT- 5 mm- arterial	Chest CECT- 5 mm- venous
EAT volume (cm^3^)	117.8 ± 57.7	87.9 ± 50.1	98.8 ± 54.1	108.6 ± 55.2	81.1 ± 47.6	90.6 ± 52.1
∆Volume, cm^3^ (%) (vs. Reference)	N/A	− 29.9 ± 10.6 (− 28.1 ± 9.5%)	− 19.0 ± 9.1 (− 17.9 ± 8.4%)	N/A	− 27.5 ± 10.7 (− 27.8 ± 8.6%)	− 18.0 ± 7.0 (− 19.4 ± 8.3%)
*p*-value 1 (vs. Reference)	N/A	< 0.001	< 0.001		< 0.001	< 0.001
EAT radiodensity(HU)	− 76.7 ± 7.3	− 75.9 ± 6.6	− 74.9 ± 5.4	− 74.0 ± 6.3	− 71.7 ± 6.0	− 72.6 ± 5.7
*p*-value 2 (vs. Reference)	N/A	0.033	0.028	N/A	< 0.001	< 0.001

Comparison of EAT volume and EAT radiodensity between standard ECG-gated cardiac NCCT with 120 kVp non-ECG gated chest NCCT image in Cohort 1, and with 100 kVp non-ECG gated chest NCCT image in Cohort 2, and between chest NCCT and contrast enhancement in arterial phase and venous phase on chest CECT in Cohort 3. Abbreviation: See Table 1

### EAT volume in Cohort 2

Using the tube voltage of 100 KPV, non-ECG-gated chest NCCT-1.25 mm EAT volume was overestimated compared to that of standard cardiac NCCT image (147.0 ± 56.6 vs. 129.6 ± 50.6 cm^3^, *p* < 0.001, see Table 3 and Fig. 2). The non-ECG-gated chest NCCT-5 mm EAT volumes (137.2 ± 53.9 vs. 129.6 ± 50.6 cm^3^, *p* < 0.001) was overestimated. Both non-ECG-gated chest NCCT EAT volumes had similar predicting value of obstructive CAD (AUC of ROC: 0.701 vs. 0.724 vs. 0.714, *p* = 0.40, Fig. 3c) and moderate to severe CAD defined by CAC score > 100 (AUC of ROC: 0.7333 vs. 0.7375 vs. 0.7333, *p* = 0.96, Fig. 3d) with cardiac NCCT EAT volumes. Furthermore, non-ECG-gated chest NCCT-1.25 mm and − 5 mm EAT radiodensity (− 83.9 ± 6.1 HU and − 79.5 ± 5.5 HU) were significantly lower (more negative) than that of cardiac NCCT (− 78.0 ± 4.7 HU), both *p*s < 0.05. The chest CT-5 mm EAT volumes were lower (Δ = − 9.5 (− 14.9, − 6.2) cm^3^, Δ% = − 6.3 (− 9.6, − 5.1) %, *p* < 0.001) than the chest CT-1.25 mm EAT volumes.

### Impact of contrast enhancement on EAT from chest CT

Chest CECT scans in arterial phase and venous phase produces EAT volumes that were consistently lower than chest NCCT [1.25 mm: (87.9 ± 50.1 and 98.8 ± 54.1 vs. 117.8 ± 57.7 cm^3^, both *p*s < 0.001), 5 mm: (81.1 ± 47.6 and 90.6 ± 52.1vs. 108.6 ± 55.2cm^3^, both *p*s < 0.001), Table 3 and Fig. 2]. Of note, EAT volumes derived from the chest venous CECT image were significantly higher than that of the chest arterial CECT image in both 1.25 mm thick and 5 mm thick images (both *p*s < 0.001). Furthermore, the chest CECT EAT radiodensities were significantly higher (less negative) than that of non-ECG-gated chest NCCT in both 1.25 mm and 5 mm (Table 3). Additionally, EAT volumes derived from the chest CECT in arterial phase were lower [1.25 mm: Δ = − 7.8 (− 10.1, − 3.9) cm^3^, Δ% = − 8.1 (− 13.1, − 4.7) %, *p* < 0.001; 5 mm: Δ = − 7.8 (− 11.1, − 5.0) cm^3^, Δ% = − 8.4 (− 14.0, − 4.9) %, *p* < 0.001)] than the counterparts derived form chest CECT in venous phase. We did not detect a significant difference of EAT radiodensity between the CECT image of the arterial phase and venous phase in 1.25 mm dataset (*p* value: 0.07), but the CECT venous EAT volumes exhibit a significant lower radiodensity (*p* value: 0.04) than the EAT volume derived from the arterial counterparts.

### Power analysis

As the sample size in each cohort was modest, we further performed the power analysis. Using an alpha level of 0.5 with two-sided test accuracy and providing the two means of EAT volume derived from reference and compared imaging data in each cohort, the null difference of the two means, the standard deviation of the difference and the sample size, the power analysis showed that the power of comparison in each cohort ranged from 0.95 to 1.00 based on the sample size of each cohort in the current study.

## Discussion

The main findings of the current study are: (1) the 120 kVp non-ECG-gated chest NCCT-1.25 mm image is an excellent alternative to the reference ECG-gated cardiac NCCT for producing accurate measurements of EAT volume; (2) a lower tube voltage of chest CT image tends to overestimate the EAT volume; (3) contrast enhancements in arterial phase and venous phase both underestimates EAT volumes in non-ECG-gated chest CT images, compared to chest NCCT EAT volumes; (4) thicker-sliced non-ECG-gated chest NCCT datasets (e.g., 5 mm) underestimate EAT volumes, compared to thinner-sliced (e.g., 1.25 mm) counterparts; (5) chest CT derived EAT volumes exhibit similar value of predicting significant coronary atherosclerosis compared to the reference EAT measurements.

To the best of our knowledge, this is the first study to systemically investigate the effects of acquisition and reconstruction parameters on EAT volume quantification in non-ECG-gated chest CT images. This is the first study to compare non-ECG-gated chest CT EAT volumes to those that were obtained using ECG-gated cardiac NCCT scans. Currently, cardiac CT scans are performed predominantly in patients with confirmed or suspected CAD [25]. Patients with chronic obstructive pulmonary disease [8, 10], breast cancer [26, 27], or lung cancer [28, 29] have a higher risk of CAD. But these patients conventionally undergo non-ECG-gated chest, but not ECG-gated cardiac, CT scans. Furthermore, chest CT scans have a much broader spectrum of clinical application [30], with 11.6 million chest CT scans in 2006 in the United States [9]. The fact that EAT volumes can be accurately estimated from chest CT scans will broaden the spectrum of EAT-associated research questions without additional radiation exposure and costly cardiac CT scanning.

### EAT volume measured in ECG-gated cardiac NCCT versus non-ECG-gated chest NCCT with controlled tube voltage

We found that the EAT volumes evaluated in non-ECG-gated chest NCCT-1.25 mm images acquired at a tube voltage of 120 kVp was almost identical to those derived from the reference cardiac NCCT (ECG-gated, 120 kVp, 3-mm slice thickness). It is reasonable to assume this similarity is a consequence of additive ECG gating and slice thickness effects. As the 3-mm slice thickness images are not routinely reconstructed in chest CT scan, we were not able to directly compare the EAT volumes between the paired cardiac and chest CT dataset with the same slice thickness. We separately discussed the effect of ECG gating and slice thickness.

ECG gating technique has been widely used to mitigate motion artifacts of coronary arteries. However, for chest CT acquisition without ECG gating, the recent technical advances—e.g. increased speed of gantry rotation and pitch, application of dual-source multidetector CT scan—shortened imaging time and substantially improved temporal resolution, further minimizing sensitivity to motion artifacts [31]. The ECG-gated cardiac CT images are typically acquired at the mid diastole to avoid large motion of cardiac ventricle, while the non-ECG gated chest CT images can be attained at any phase of the cardiac cycle such as late systole or mid diastole. A recent cardiac CECT study that compared EAT volumes at late systole and mid diastole concluded that the EAT volume is independent of the cardiac phase [32]. A previous study has further demonstrated that the pericardial volume (the total volume within pericardium) and the heart volume (the volume of the myocardium and the cardiac chambers) have parallel changes over the cardiac cycle [33], which means the difference between the two volumes (i.e., EAT volume) are relatively consistent during the cardiac cycle. Therefore, we believe that the absence of ECG gating has no significant impact on EAT volume quantification in chest CT image. Lu et al. reported a minimal benefit of ECG gating in contrast-enhanced CT images for measuring the right ventricular volume [34]. Several studies evaluated the effect of absent ECG gating in measuring coronary calcium score and consistently demonstrated a close association with that by ECG-gated CT [19, 35, 36]. Consequently, the effect of ECG-gating is insignificant in gross and fine structural measurements. Although Lee et al. reported that the EAT area measured only in one slice of non-gated CT image exhibited a good correlation with cardiac CECT-derived EAT area [6], EAT evaluated from a single slice is insufficient to represent the whole EAT volume. Nagayama et al. compared the EAT volume derived from non-gated chest NCCT with that from the cardiac CECT in 117 patients and reported an excellent correlation and similar predicting value of CAD between the two measurements [7]. However, non-gated chest CT was not compared with cardiac NCCT, as the latter has been more commonly used to quantify the EAT volume, and cardiac CECT might not be acquired due to radiation exposure and cost consideration. The previous studies [6, 7] only identified the excellent correlation between the non-gated chest CT-derived EAT area/volume and cardiac CECT-derived EAT area/volume. However, our study identified that the 120 kVp non-ECG-gated chest NCCT-1.25 mm images were an excellent alternative to the standard cardiac NCCT to the actual EAT volumetric measurement with the prerequisite that the radiodensity threshold of − 190 to − 30 HU was not adjusted.

Christensen et al. reported that 5-mm thick chest NCCT underestimated the coronary calcium score when compared with that derived from 1.25-mm and 2.5-mm chest NCCT[18]. However, the effect of slice thickness on EAT volume quantification has not been explored yet. Although EAT predominantly contains fat, there are abundant small vessels, nerves, and immune cells [1] which present much higher radiodensities + 40 to + 60 HU [37]. Thicker slices, and thus larger voxel dimensions, increase the potential for partial volume errors, where a higher fraction of the voxel is occupied by material with different radiodensities. Consequently, the NCCT images with thicker slices have a smaller percentage of voxels fall into the radiodensity range of − 190, − 30 HU. Furthermore, our study observed the EAT radiodensities of non-ECG-gated chest NCCT-1.25 mm scans were similar to the standard cardiac NCCT but lower than those of chest CT-5 mm scans. This may explain a smaller EAT volumes in chest CT-5 mm images without additional radiodensity threshold adjustments. And chest CT-5 mm images consistently produced a lower EAT volume than that from the 1.25 mm counterpart images in three cohorts.

### Tube voltage and EAT volume

Compared to the standardized cardiac NCCT EAT volume, which is similar to the non-ECG-gated chest NCCT-1.25 mm EAT volumes with tube voltage of 120 kVp, the chest NCCT-1.25 mm EAT volumes with tube voltage of 100 kVp were significantly overestimated. Consequently, a lower tube voltage contributed to the overestimation of EAT volume. Additionally, we also observed that lowering the tube voltage decreases the EAT radiodensity (i.e., more negative). Similarly, lower attenuation values (more negative) of pericardial fat from a lower tube energy scan have been previously reported [38]. It is possible that lower EAT radiodensity values contributed to overestimation of EAT volumes, and different radiodensity thresholds are optimal for EAT volume quantification at different tube energies. Furthermore, a lower tube voltage reduces image signal to noise ratio, which may also impact the accuracy of EAT estimation [39]. Marwan et al. also elegantly investigated the effect of tube voltage of quantifying EAT volume from cardiac NCCT images in a bigger cohort of 127 patients and found a significant overestimation of the EAT volume using lower tube voltage [39]. However, this study primarily targeted ECG-gated cardiac CT. Cardiac CT scan is commonly standardized at the tuber voltage of 120 kVp, while the tube voltage of the chest CT substantially varies, especially in the low-dose CT, to reduce the radiation exposure when repeated scans are required. Consequently, investigating the influence of tube voltage in chest CT is necessary and might have more clinical value. Our study is the first to investigate the effect of tube voltage in non-ECG gated CT images and had a similar result to Marwan et al. study that a lower tube voltage (i.e., 100 kPv) overestimated the EAT volume by ~ 17 cm^3^ (vs. ~ 14 cm^3^ in Marwan et al. study) when compared to the standard tube voltage (i.e., 120 kPv). Compared to the previous study [39], although ECG gating was absent in the current study might, we believe the corresponding impact is trivial based on our earlier discussion.

### Contrast agent and EAT volume

Although in our previous study[17] and the study by Marwan et al., [39], EAT volumes derived from ECG-gated cardiac arterial-phase CECT datasets have been demonstrated to be significantly smaller than those derived from the cardiac NCCT datasets when using the standard radiodensity threshold (− 190, − 30) HU [17], the current study is the first to confirm that contrast agents in the arterial phase and venous phase both underestimates the EAT volume in non-ECG-gated chest CT, but with a various degree, regardless of slice thicknesses (i.e., 5 mm and 1.25 mm). In the current study, arterial-phase enhancement in chest CT with the absence of ECG gating, compared to the chest NCCT images, underestimated the EAT volume by ~ 30 cm^3^, which were similar to our previous study [17] by ~ 34 cm^3^, and Marwan et al. study [39] by ~ 31 cm^3^ in cardiac CT images. This again demonstrated that the ECG gating does not affect the effect of contrast enhancement on EAT volume measurement. Although we observed that adding a contrast agent increases the EAT radiodensity (less negative) in chest CT scans, the degree of increased radiodensity was not parallel with the underestimated EAT volume from chest NCCT to chest CECT. Potentially, a higher level of contrast and image noise of chest NCCT, compared to the chest CECT, may overestimate the contour of EAT.

The venous-phase enhanced chest CT images have been reported to have incremental value over arterial-phase enhancement for diagnostic purpose [40] and are increasingly acquired in clinical practice. However, the difference in EAT volume quantification between the venous-phase enhancement and the conventional arterial-phase enhancement has never been explored. We are the first to investigate the effect of the contrast agent in venous phase, and the EAT volume derived from the venous CECT image was significant higher, as well as a lower EAT radiodensity than the counterparts derived from the arterial CECT image under the condition of fixed tube voltage, slice thicknesses and other imaging setting. This finding indicates that a mixed used of contrast-enhanced CT image in different vascular phases are inappropriate. However, a higher chest CECT EAT radiodensity may not be enough to explain a significantly lower chest CECT EAT volume than the EAT volume derived from the chest NCCT, and other factors may be involved.

### EAT volume and CAD

We observed that non-ECG-gated chest CT EAT volumes, regardless of the acquisition and reconstruction parameters, and the standard cardiac NCCT counterparts have a similar predicting value of significant coronary atherosclerosis. It is likely that EAT volumes from chest CT scans can be widely applicable to predict CAD severity and adverse outcomes, if the same imaging acquisition protocol is applied to all subjects in a study. On the other hand, a mixed use of chest CT dataset, with inconsistent tube voltages, slice thicknesses, and use of contrast agent, is not recommended.

### Limitations

First, although thousands of CT dataset have been reviewed in our clinical database, only a moderate sample size with paired cardiac and chest CT scans were identified; however, the power analysis has demonstrated that our sample size enables sufficient power based on our result. Second, the investigation of the tube voltage would ideally be explored in paired chest CT datasets with different tube voltages, which were not available in this retrospective study. However, in Cohort 2, standard cardiac NCCT EAT volume was an excellent alternative of the 120 kVp non-ECG-gated chest NCCT 1.25 mm EAT volume, which enables an indirect comparison of chest CT image of two tube voltages. Third, in Cohort 3, although the chest CECT derived EAT exhibited a higher radiodensity than those derived from the chest NCCT, the degree of the difference of EAT radiodensity did not seem to be parallel with the difference of the EAT volumes, and other factors might contribute to the difference of the EAT volumes. Finally, the impact of interslice gap on EAT volume quantification should be further investigated in future studies.

## Conclusion

The 120 kVp non-ECG-gated chest NCCT-1.25 mm image is an excellent alternative of the standard cardiac NCCT image to measure EAT volume. Under the condition that a consistent image acquisition and reconstruction setting is used in the chest CT image for the EAT quantification, all chest CT-derived EAT volumes are adequate alternatives of the standard cardiac NCCT image to study the association of EAT volume and coronary artery disease. And a mixed used of chest CT image is not recommended. Consequently, EAT volume quantification can be tremendously expanded from cardiac NCCT image to a broad spectrum of chest NCCT datasets. The early risk stratification of cardiovascular risk and outcome via measuring EAT volume can be performed in a remarkably more enormous scope and amount of chest CT images in a wider variety of patients with non-coronary diseases or non-cardiac diseases and not undergoing cardiac CT scans.

## Supplementary Information

**Additional file 1: Table S1.** Clinical indications for CT scan in three cohorts

## Data Availability

The datasets generated during and analyzed during the current study are not publicly available due to the privacy of individuals that participated in the study but are available from the corresponding author on reasonable request.

## Abbreviations

EAT: Epicardial adipose tissue
NCCT: Non-contrast computed tomography
ECG: Electrocardiogram
CECT: Contrast-enhanced CT images
HU: Hounsfield unit
CAD: Coronary artery disease
AUC: Area under the curve
ROC: Receiver operating characteristic
