# Supplementary material for: Comparison of epicardial adipose tissue volume quantification between ECG-gated cardiac and non-ECG-gated chest computed tomography scans
Source: BMC Cardiovasc Disord. 2022 Dec 13;22:545. doi: 10.1186/s12872-022-02958-2 (PMC9746017; doi:10.1186/s12872-022-02958-2)
Supplement: Supplementary file 1 — Additional file 1: Table S1. Clinical indications for CT scan in three cohorts [file 12872_2022_2958_MOESM1_ESM.docx]

Supplemental Table 1. Clinical indications for CT scan in three cohorts

|  | Cohort 1 (*N* = 49)  Time interval of 2 CT scans: < 2 weeks | | Cohort 2 (*N* = 34)  Time interval of 2 CT scans: < 2 weeks | | Cohort 3 (*N* = 32) |
| --- | --- | --- | --- | --- | --- |
| CT dataset | ECG-gated Cardiac NCCT | non-ECG-gated Chest  NCCT | ECG-gated Cardiac NCCT | non-ECG-gated Chest  NCCT | non-ECG-gated Chest NCCT & CECT |
| Chest pain | 61.9% | 34.5% | 55.6% | 33.3% | 16.7% |
| Elevated T wave | 9.5% |  | 11.1% |  |  |
| preoperative assessment for cancer-related surgery | 23.8% | 23% | 22.2% | 16.7% | 27.8% |
| Infection |  | 11.5% |  | 22.2% | 22.2% |
| Chronic obstructive pulmonary disease |  | 11.5% |  | 11.1% | 5.6% |
| Lung nodes in x-ray |  | 11.5% |  | 5.6% | 16.7% |
| others | 4.8% | 8% | 11.1% | 11.1% | 11% |
